# Baseline measures of cerebral glutamate and GABA levels in individuals at ultrahigh risk for psychosis: Implications for clinical outcome after 12 months

**DOI:** 10.1192/j.eurpsy.2020.77

**Published:** 2020-08-07

**Authors:** C. Wenneberg, B. Y. Glenthøj, L. B. Glenthøj, B. Fagerlund, K. Krakauer, T. D. Kristensen, C. Hjorthøj, R. A. E. Edden, B. V. Broberg, K. B. Bojesen, E. Rostrup, M. Nordentoft

**Affiliations:** 1 Copenhagen Research Center for Mental Health, University of Copenhagen, Copenhagen, Denmark; 2 Center for Neuropsychiatric Schizophrenia Research, CNSR, and Center for Clinical Intervention and Neuropsychiatric Schizophrenia Research, CINS, Mental Health Centre Glostrup, University of Copenhagen, Copenhagen, Denmark; 3 Functional Imaging Unit, FIUNIT, Department of Clinical Physiology, Nuclear Medicine and PET, University of Copenhagen, Copenhagen, Denmark; 4 Department of Public Health, University of Copenhagen, Copenhagen, Denmark; 5 Russell H. Morgan Department of Radiology and Radiological Science, The Johns Hopkins University School of Medicine, F.M. Kirby Research Center for Functional Brain Imaging, Kennedy Krieger Institute, Baltimore, Maryland, USA

**Keywords:** ^1^H-MRS, GABA, glutamate, outcome, prodromal, UHR

## Abstract

**Background.:**

Cerebral glutamate and gamma-aminobutyric acid (GABA) levels might predict clinical outcome in individuals at ultrahigh risk (UHR) for psychosis but have previously primarily been investigated in smaller cohorts. We aimed to study whether baseline levels of glutamate and GABA in anterior cingulate cortex (ACC) and glutamate in thalamus could predict remission status and whether baseline metabolites differed in the remission versus the nonremission group. We also investigated the relationship between baseline metabolite levels and severity of clinical symptoms, functional outcome, and cognitive deficits at follow-up.

**Methods.:**

About 124 UHR individuals were recruited at baseline. In this, 74 UHR individuals were clinically and cognitively assessed after 12 months, while remission status was available for 81 (25 remission/56 nonremission). Glutamate and GABA levels were assessed at baseline using 3 T proton magnetic resonance spectroscopy. Psychopathology, symptom severity, and remission were assessed with the Comprehensive Assessment of At-Risk Mental States and Clinical Global Impression and functional outcome with the Social and Occupational Functioning Assessment Scale. Cognitive function was estimated with the Cambridge Neuropsychological Test Automated Battery.

**Results.:**

There were no differences between baseline glutamate and GABA levels in subjects in the nonremission group compared with the remission group, and baseline metabolites could not predict remission status. However, higher baseline levels of GABA in ACC were associated with clinical global improvement (*r* = −0.34, *N* = 51, *p* = 0.01) in an explorative analysis.

**Conclusions.:**

The variety in findings across studies suggests a probable multifactorial influence on clinical outcome in UHR individuals. Future studies should combine multimodal approaches to attempt prediction of long-term outcome.

## Introduction

The initial psychosis prodrome is seen as a forerunner for a first episode of psychosis and possibly the development of schizophrenia. This period is accompanied by a gradual deterioration in psychosocial functioning and the development of clinical symptomatology. Studies have focused on identifying putative prodromal individuals by developing clinical criteria to help detect individuals at ultrahigh risk (UHR) of developing psychosis, such as The Comprehensive Assessment of at-Risk Mental States (CAARMS). CAARMS requires the presence of subthreshold psychotic symptoms or a genetic predisposition along with a loss of psychosocial function [1,2]. A meta-analysis of UHR studies until 2011 concluded that 27% of UHR individuals transitioned to a psychotic disorder within 2 years [3]. However, since more recent studies indicate a decline in transition rates (8–28% within 1 year) compared with earlier findings [4,5], stratification of UHR individuals into the subgroups transition, nonremission, and remission has been suggested as more relevant than solely focusing on transition or nontransition [4,6,7].

Studies on glutamatergic and GABAergic neurotransmission point to a possible role in the pathophysiology of schizophrenia [8–12] with hypofunction of the N-methyl-*D*-aspartate glutamate receptor and consequently aberrant glutamate release in target regions as a central theory [8,13,14]. An increasing amount of research is likewise investigating whether glutamate and gamma-aminobutyric acid (GABA) abnormalities are present prior to the development of frank psychotic symptoms [8,11,15,16]. Recent meta-analyses have suggested either increased levels of glutamate in most cerebral regions [11] or decreased thalamic glutamate levels but no significant differences in glutamate or Glx between UHR individuals and healthy controls in remaining brain regions [16]. Studies on GABA levels in UHR states are still limited, but meta-analytical studies propose that there is no difference between neither established schizophrenia or UHR and healthy controls [12,16]. Some studies do, however, report differences in glutamate or Glx [7,17–23] as well as GABA [21,23,24] levels between UHR and healthy controls, although results are diverging regarding the direction of the differences. This suggests that aberrant metabolite levels may only be present in the subgroup of actual prodromal patients, supported by studies showing that the degree of glutamatergic dysfunction might predict clinical outcome [6,19,25]. Although not previously investigated in a sizeable sample, studies have linked aberrant levels of glutamate to poor clinical [7,26] and functional outcome [26,27].

Furthermore, abnormal levels of glutamate (increased as well as decreased) have been found to be associated with the severity of attenuated psychotic symptoms [7,21,28], negative symptoms [29], and poor performance on neurocognitive tests of visuospatial attention [22] and working memory [21]. Similarly, lower levels of GABA have been associated with more severe negative symptoms [30]. We have previously—in the same cohort as investigated in this paper, comprising 122 UHR individuals and 60 healthy controls at baseline [31]—found negative correlations in the anterior cingulate cortex (ACC) in the UHR group between baseline Glx levels and CAARMS composite score and between GABA and alogia. Moreover, likewise in ACC, we found a number of associations between metabolite levels and cognitive performance on subtests from the Cambridge Neuropsychological Test Automated Battery (CANTAB) [32], that is, a positive correlation between performance on a spatial working memory (SWM) task (SWM—between errors) and higher levels of glutamate and Glx, as well as better performance on a set-shifting task (Intra-Extra Dimensional Set Shift—Total errors [adjusted]) and higher levels of glutamate, Glx, and GABA. In thalamus, we found a positive association between glutamate and attention (Rapid Visual Information Processing A’). Since we found no associations in the healthy control group, this could indicate that disturbances of the normal relationship are present in the UHR group—supported by similar findings in other studies [33,34]—suggesting that changes in glutamate and GABA may accompany changes in clinical and cognitive status at follow-up. Considering that risk of transitioning to a psychotic disorder has been shown to be higher for UHR individuals experiencing more attenuated positive symptoms, more severe negative symptoms, and lower level of functioning [4], investigating the relations between clinical symptoms, cognitive deficits, functional outcome, and metabolite levels in longitudinal studies with a large study sample and hence sufficient power to detect the statistically significant associations could provide new knowledge in the field or possibly replicate previous findings of the relation between glutamate/GABA and the development of psychosis [7,26,35].

In this longitudinal study, we examined the relationship between functional, clinical, and cognitive outcomes after 12 months and baseline levels of glutamate, Glx, and GABA in UHR individuals.

The primary aim of the study was to determine whether baseline levels of glutamate, Glx, and GABA in ACC and glutamate levels in thalamus differed between UHR groups with remission and nonremission+transition (nonremission+) after 12 months. The secondary aim was to determine whether baseline levels of glutamate, Glx, and GABA were predictive of clinical and functional outcome and sustained cognitive deficits after 12 months. Based on the available literature [7,26,36] and our previous work [16,31], where lower levels of glutamate and GABA were connected to worse clinical symptoms and worse performance on cognitive tests at baseline, we hypothesized that the nonremission+ group would have lower levels of glutamate, Glx, and GABA than the remission group. We also hypothesized that lower levels of glutamate, Glx, and GABA at baseline would be associated with worse functional and clinical outcome as well as sustained cognitive deficits.

## Methods

### Participants

Participants were recruited between March 2014 and December 2017 as part of a randomized clinical trial, the FOCUS trial [37]. The study was approved by the Committee on Biomedical Research Ethics (H-6-2013-015) and The Danish Data Protection Agency. All participants provided informed consent before inclusion in the study. The baseline sample of the ^1^ proton magnetic resonance spectrum (1H-MRS) study consisted of 122 help-seeking individuals aged 18–40 years from in- and outpatient facilities in Copenhagen, all meeting standardized at-risk criteria of the instrument CAARMS (attenuated psychotic symptoms, brief limited intermittent psychotic symptoms, and/or trait and vulnerability group). Exclusion criteria (e.g., a psychotic episode of more than 1 week’s duration, physical illness with psychotropic effect, autistic disorders, IQ < 70, or attenuated psychotic symptoms caused by acute intoxication), as well as inclusion criteria for healthy controls, are described in detail previously [31].

At baseline, the MRS study sample consisted of 122 UHR individuals and 60 healthy controls (the FOCUS trial included a total of 146 UHR individuals and 70 healthy controls). A total of 74 UHR individuals completed follow-up assessments at 12 months (follow-up data for healthy controls are not part of the present study). Of the 48 UHR individuals lost to follow-up, remission status was obtainable from clinical records for 7 additional individuals, resulting in a total of 81 UHR individuals for the present study on remission status.

### Clinical and cognitive assessments

Participants completed clinical assessments as well as cognitive testing at follow-up. Symptomatology was assessed with CAARMS (unusual thought content, nonbizarre ideas, perceptual abnormalities, disorganized speech, and a calculated CAARMS composite score), the Scale for the Assessment of Negative Symptoms (SANS) [38], and the Brief Psychiatric Rating Scale Expanded Version (BPRS-E) [39]. Level of functioning was assessed using the Social and Occupational Functioning Assessment Scale (SOFAS) [40] and symptom severity with the Clinical Global Impression (CGI) scale [41]. Four measures of cognitive performance from CANTAB were included in this study based on our baseline findings: SWM (outcomes were SWM strategy and SWM between errors), rule acquisition, reversal tested with intra-extra dimensional set shift (outcome was IED total errors [adjusted]), and sustained visual attention tested with rapid visual information processing (outcome was RVP A’).

In testing the hypothesis that baseline glutamate, Glx, and GABA levels were associated with clinical outcome, UHR subjects were divided into remission and nonremission groups based on the follow-up clinical assessment CAARMS scores. The nonremission+ group included UHR individuals who still met the CAARMS criteria for being at UHR as well as individuals who had transitioned to a psychotic disorder (established by CAARMS or by receiving a diagnosis on the psychosis spectrum). The remission group included individuals who no longer fulfilled the CAARMS UHR criteria.

### ^1^ H-MRS

Scans were performed at baseline using a 3T Philips Achieva system as previously described [31]. Glutamate and Glx levels were obtained using point-resolved spectroscopy (PRESS) (TR 3,000 ms, TE 30 ms, 128 averages with MOIST water suppression), and ^1^H-MRS voxels were placed in ACC (2.0 x 2.0 x 2.0 cm, Brodmann area 24 and 32) and in left thalamus (2.0 x 1.5 x 2.0 cm). Levels of GABA were obtained using the Mescher–Garwood point resolved spectroscopy (MEGA-PRESS) sequence (TE = 68 ms; TR = 2,000 ms, 14 ms editing pulse applied at 1.9 ppm and 7.5 ppm, 320 averages with MOIST water suppression) in a voxel placed in the ACC (3.0 x 3.0 x 3.0 cm). Metabolite levels used for this paper are the baseline in vivo water-scaled values corrected for partial volume cerebrospinal fluid contamination as described in detail previously [31], where further details of data acquisition and analysis are likewise described.

### Statistical analyses

Group differences in demographic and clinical variables, cognitive tests, and metabolite levels were assessed using two-tailed independent samples *t*-tests, Mann–Whitney *U*, or Pearson chi-square as appropriate.

We analyzed data with incomplete variables by excluding all incomplete cases (complete-case analysis). Since analysis of complete records may produce results substantially different from those obtained if a full sample were available, multiple imputation [42] was applied to account for missing data at follow-up under the assumption that data were missing at random. Multiple imputation analyses did not yield results that differed significantly from the results obtained by complete-case analyses. Hence, the complete-case analyses are reported in this paper.

Besides examining differences in metabolite levels between the remission and nonremission+ group, exploratory subgroup analyses were done to test for significant differences in baseline metabolite levels between the remission, nonremission, and transition group.

The predictive value of glutamate, Glx, and GABA in determining the clinical outcome (remission or nonremission+) was assessed using binomial logistic regression. An exploratory multinomial logistic regression was performed to further assess the predictive effects of glutamate, Glx, and GABA combined with baseline CAARMS composite score on the likelihood of an outcome of transition, nonremission, or remission. Explorative analyses of correlations between baseline metabolite levels and follow-up measures of SOFAS and CGI Global improvement were run to investigate possible associations to functional outcome.

Partial correlations were run separately for each metabolite to determine relationships between glutamate, Glx, and GABA levels at baseline and CAARMS composite and SANS global at follow-up to test whether lower glutamatergic and GABAergic levels at baseline were associated with worse clinical symptoms at follow-up. Correlations between metabolite levels and absolute change in CANTAB subtest scores from baseline to follow-up were likewise calculated using partial correlation, covarying for baseline scores to control for regression to the mean.

All metabolites were normally distributed. When clinical or cognitive measures were not normally distributed, nonparametric partial correlations were run. Controlling for factors that have been shown to influence metabolite levels (age [43,44], gender [45], tobacco smoking [46], antipsychotic medication [47,48], and gray matter/white matter ratio [49]) in all analyses did not change the significance.

Subsequent analyses were run excluding individuals with current use of benzodiazepines, antipsychotics, or any form of substance abuse, respectively. This did not change the results reported.

Statistical analyses were performed in SPSS version 25 (SPSS, Chicago, IL, USA).

## Results

### Demographics, clinical, and cognitive variables

About 25 UHR individuals had remitted from UHR status and no longer fulfilled the CAARMS criteria. The remaining 56 UHR individuals had nonremission, 43 still fulfilled the UHR criteria, and 13 individuals had a confirmed transition to a psychotic disorder (resulting in a known transition rate of 16% after 12 months). The sample characteristics at 12-month follow-up are shown in Table 1. The remission group was significantly better educated than the nonremission group (controlling for this did not influence results), but no other baseline demographic variables differed between the remission and nonremission group. At follow-up, the nonremission groups presented with significantly worse positive symptoms, avolition, and level of functioning and less global clinical improvement. There were no differences in baseline demographic, clinical, or cognitive measures between UHR individuals that are included in this study and those that are lost to follow-up (data not shown).

**Table 1.** Demographics, clinical characteristics, and cognition at 12-months follow-up for UHR remission and non-remission groups

Variable	Remission (N = 25)	Non-remission (N = 56)		Statistics	P-values
**Gender** (N)					
Female/Male	16/9	26/30		χ^2^ = 2.14	0.16
**Age at baseline, years** ±SD	25.2 ±2.7	24.5 ±5.0		U = 548, Z = -1.56	0.12
**Parental SES** (%)					
(High/moderate/low/missing)	56.0/36.0/0.0/8.0	46.4/32.1/16.1/5.4		χ^2^ = 4.45	0.12
**Education, years at baseline** ±SD	15.6 ±1.9	14.3 ±3.2		U = 428, Z = -2.80	0.01*
**Cannabis smoking last year** (N)					
Yes/No/Missing	7/18/0	12/37/7		χ^2^ = 0.11	0.78
**Antipsychotic treatment** (N)					
Antipsychotic naïve at baseline (no/yes/missing)	14/11/0	26/29/1		χ^2^ = 0.52	0.63
Current antipsychotic (yes/no/missing)	11/13/1	26/21/9		χ^2^ = 0.57	0.47
**CAARMS subgroups at baseline** (%)					
Vulnerability APS	12.0 100.0	25.0 100.0		χ^2^ = 1.76 -	0.24
BLIPS	0.0	2.0		U = 675, Z = -0.95	0.57
	**Baseline**	**12-month follow-up**			
**CAARMS composite score** ^**¤****#**^ ±SD	(N = 122) 50.4 ±14.3	(N = 25) 13.6 ±9.1	(N = 49) 37.8 ±12.7	U = 72, Z = -6.19	< 0.001**
**SANS** ^**#**^ Affect Alogia Avolition Anhedonia	(N = 117) 1.4 ±1.0 1.2 ±1.0 1.7 ±0.9 1.7 ±1.2	(N = 24) 1.4 ±1.1 0.9 ±1.0 1.2 ±1.0 1.0 ±1.0	(N = 49) 1.4 ±1.1 1.0 ±1.1 1.6 ±1.0 1.5 ±1.8	χ^2^ = 1.15 χ^2^ = 1.00 χ^2^ = 8.32 χ^2^ = 5.61	0.95 0.84 0.04* 0.36
**BPRS total** ^**#**^	(N = 117) 42.3 ±0.7	(N = 24) 31.2 ±4.5	(N = 49) 42.0 ±6.5	T = 7.15	< 0.001**
**SOFAS** ±SD	(N = 121) 55.4 ±11.0	(N = 25) 64.6 ±10.8	(N = 49) 58.2 ±10.0	U = 401, Z = -2.43	0.01*
**CGI Global improvement** ^**#**^ ±SD		(N = 21) 2.52 ±0.24	(N = 38) 3.18 ±0.20	U = 256, Z = -2.35	0.02*
**CANTAB** ±SD RVP A’ SWM Strategy^#^ SWM Between errors^#^ IED Total errors (adjusted)^#^	(N = 117) 0.9 ±0.1 27.1 ±6.3 10.5 ±10.0 19.7 ±17.1	(N = 24) 0.9 ±0.1 26.2 ±6.7 9.3 ±15.4 13.1 ±14.8	(N = 46) 0.9 ±0.0 23.8 ±6.0 5.2 ±8.2 13.0 ±12.3	U = 537, Z = -0.04 U = 467, Z = -1.06 U = 467, Z = -1.08 U = 495, Z = -0.57	0.97 0.29 0.28 0.57

Abbreviations: UHR, Ultra-high risk; SD, Standard deviation; SES, Socio-economic status; CAARMS, The comprehensive assessment of at risk mental states; APS, Attenuated psychotic symptoms; BLIPS, Brief limited intermittent psychotic symptoms; SANS, The scale for the assessment of negative symptoms; BPRS, The brief psychiatric rating scale; SOFAS, Social and occupational function assessment scale; CGI, Clinical global impression scale; CANTAB, Cambridge neuropsychological test automated battery; RVP, Rapid visual information processing; SWM, Spatial working memory; IED, Intra-extra dimensional set shift

*p < 0.05; **p < 0.01; ^#^a lower score is better.

¤ CAARMS composite score was calculated according to the formula: (I utc * F utc) + (I nbi *F nbi) + (I pa * F pa) + (I ds * F ds). I, intensity; utc, unusual thought content; F, frequency; nbi, non-bizarre ideas; pa, perceptual abnormalities; ds, disorganized speech

### Glutamate and GABA levels and the relation to remission and nonremission

**Figure 1.**
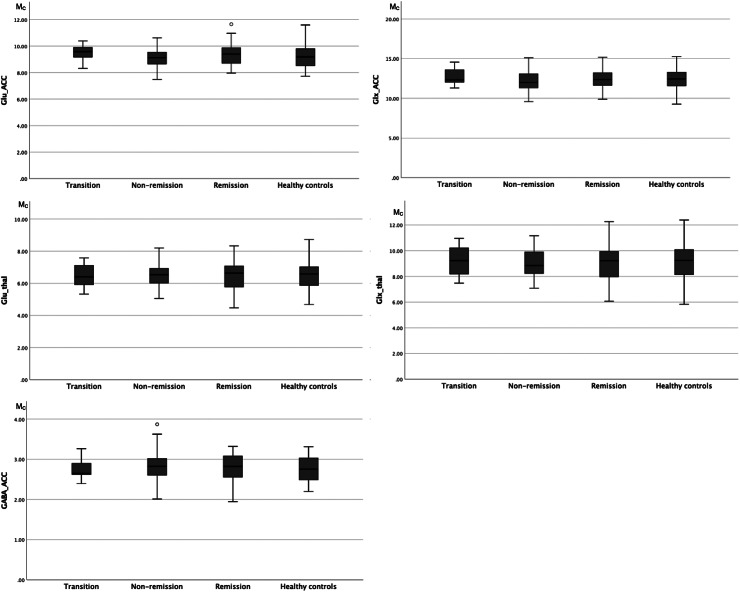
Distribution of glutamate, Glx, and GABA in ACC and thalamus at baseline in healthy controls, remission, nonremission, and transition groups determined at 12-month follow-up. Abbreviations: ACC, anterior cingulate cortex; GABA, gamma-aminobutyric acid; Glu, glutamate; Glx, glutamate + glutamine; Mc, metabolite concentrations corrected for partial volume cerebrospinal fluid contamination; thal, thalamus.

The mean water corrected levels of glutamate, Glx, and GABA at baseline are provided in Table 2A,2B. Baseline metabolite levels did not differ significantly between the remission and nonremission group for any metabolite levels in neither ACC nor thalamus. For illustrative purposes, the distribution of glutamate, Glx, and GABA at baseline in healthy controls, remission, nonremission, and transition, respectively, can be seen in Figure 1.

**Table 2A.** Water corrected mean metabolite levels at baseline in ACC and thalamus in UHR with remission or non-remission at 12-months follow-up, respectively

	ACC				Left thalamus		
	Remission	Non-remission+	P-value		Remission	Non-remission+	P-value
**Glu**	9.39 ±0.89 (N = 25)	9.20 ±0.74 (N = 54)	0.33		6.49 ±1.10 (N = 22)	6.51 ±0.70 (N = 47)	0.93
**Glx**	12.46 ±1.27 (N = 25)	12.29 ±1.22 (N = 54)	0.60		9.13 ±1.55 (N = 22)	9.10 ±1.11 (N = 47)	0.87
**GABA**	2.18 ±0.25 (N = 24)	2.17 ±0.23 (N = 46)	0.90		-	-	-

Abbreviations: ACC, Anterior cingulate cortex; UHR, Ultra-high risk; Glu, Glutamate; Glx, glutamate+glutamine; GABA, Gamma-aminobutyric acid

**Table 2B.** Water corrected mean metabolite levels at baseline in ACC and thalamus in UHR with remission, non-remission, or transition group at 12-months follow-up

	ACC					Left thalamus			
	Remission	Non-remission	Transition	One-Way ANOVA		Remission	Non-remission	Transition	One-Way ANOVA
**Glu**	9.39±0.89 (N = 25)	9.11±0.76 (N = 41)	9.47±0.60 (N = 13)	0.22		6.49±1.09 (N = 22)	6.50±0.70 (N = 36)	6.52±0.75 (N = 11)	0.99
**Glx**	12.46±1.27 (N = 25)	12.17±1.27 (N = 41)	12.71±1.03 (N = 13)	0.35		9.13±1.55 (N = 22)	9.03±1.06 (N = 36)	9.23±1.28 (N = 11)	0.89
**GABA**	2.18 ±0.25 (N = 24)	2.20 ±0.23 (N = 35)	2.21 ±0.23 (N = 11)	0.57		-	-	-	-

Abbreviations: ACC, Anterior cingulate cortex; UHR, Ultra-high risk; Glu, Glutamate; Glx, glutamate+glutamine; GABA, Gamma-aminobutyric acid

Neither glutamate (*χ^2^*(1) = 0.996, *p* = 0.32), Glx (*χ^2^*(1) = 0.281, *p* = 0.60), or GABA (*χ^2^*(1) = 0.016, *p* = 0.90) levels in ACC nor glutamate (*χ^2^*(1) = 0.012, *p* = 0.91) or Glx (*χ^2^*(1) = 0.026, *p* = 0.87) levels in thalamus were found to be predictive of remission at 12 months in the binomial regression analyses. The exploratory multinomial logistic regression found a trend level association between higher baseline glutamate levels in ACC and remission (*χ^2^*(1) = 4.06, *p* = 0.06) based on a prediction model also including CAARMS composite score at baseline (predictive value of CAARMS (*χ^2^*(1) = 20.04, *p* < 0.005) and of glutamate (*χ^2^*(1) = 1.78, *p* = 0.18). No significant prediction models were found, and no predictors were individually significant.

### Glutamate and GABA levels and associations to clinical and cognitive outcome

Glutamate in ACC was positively correlated to the change in SWM between errors (*r* = 0.25, *N* = 69, *p* = 0.04), but when controlling for baseline score, this association was no longer significant.

**Figure 2.**
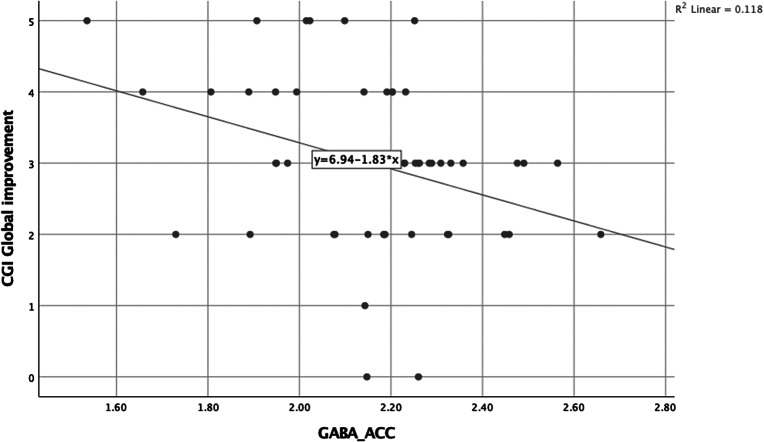
As an exploratory finding, higher GABA levels in ACC were related to clinical improvement at 12 months. Abbreviations: ACC, anterior cingulate cortex; CGI, Clinical Global Impression; GABA, gamma-aminobutyric acid.

For the entire UHR group, we found baseline levels of GABA in ACC to be negatively associated with CGI global improvement (a lower score is better) (*r* = −0.34, *N* = 51, *p* = 0.01) (**Figure 2**).

No other significant associations were found between baseline metabolite levels and cognitive or functional outcomes. We found no associations with any clinical outcome measure.

## Discussion

In the present study, we investigated whether UHR individuals with poor clinical outcome were characterized by baseline disturbances of glutamate, Glx, and GABA levels compared to remitters, and we explored the association between baseline metabolite levels and sustained cognitive deficits after 12 months. We hypothesized that baseline levels of glutamate, Glx, and GABA would predict remission from the UHR state, and that metabolite levels would differ between the remission and nonremission group. We found no support for these hypotheses in the present study. We did find that a multinomial prediction model including baseline levels of glutamate in ACC and CAARMS composite score trended toward higher levels of glutamate predicting remission rather than nonremission, which could support previous findings showing low thalamic glutamate levels at baseline to be related to nonremission of symptoms and a progressive worsening of symptoms over time as well as lower glutamate levels in ACC at follow-up than at baseline (although not related to clinical outcome) [7]. Since we found no group-level differences in baseline metabolite levels between the remission and nonremission group and did not find any single metabolite to be predictive of clinical, functional, or cognitive outcomes, this suggests that—if present—metabolite perturbances are subtle and possibly only present in part of the UHR group. This does, however, contrast findings in a first episode cohort finding higher levels of glutamate to be related to short-term lack of remission [50], which could be explained by some studies suggesting that glutamatergic disturbances originate in thalamus and are different as the illness progresses from UHR stages to established schizophrenia [51].

As an exploratory finding, higher GABA levels in ACC were related to clinical improvement rated by CGI (although an explorative measure, the significance would not survive correction for multiple comparisons [thresholded *p* < 0.01 for eight outcome measures]). We found no other metabolite relations to clinical scores. Previously, lower levels of glutamate have been associated to low level of functioning [7], severity of attenuated psychotic symptoms [7,21] as well as to negative symptoms [29]. The opposite association has been found by others with increased levels of glutamate associating to severity of attenuated psychotic symptoms [28], poor functional outcome [26], and transition to psychosis [26]. While some have found no association between metabolite levels and clinical symptoms [22], one study found lower levels of GABA in medial prefrontal cortex to be associated with more severe negative symptoms [30].

Our baseline findings suggested that higher metabolite levels associated with better performance on some cognitive tests. In this study, we did not find proof that these associations were maintained over time since absolute changes in test scores from baseline to 12-months follow-up did not significantly associate with baseline metabolite measures. To our knowledge, longitudinal changes in neurocognitive function and the relation to glutamate and GABA have not previously been investigated, and the associations to neurocognitive deficits might not be robust over time. It could be speculated that the loss of a normal relationship between metabolite levels and cognitive function could still be present in the subgroup of UHR individuals with the poorest outcome. We did, however, on an explorative note, not find any subgroup associations based on remission status and no difference in cognitive performance between groups. Nevertheless, cognitive deficits at follow-up could still be associated with lower follow-up levels of metabolites, which we have not obtained for the present study.

We found a remission rate of 31% and a transition rate of 16%. The latter is in line with declining transition rates across cohorts, while the remission rate in the present study is slightly lower than reported previously [52]. This might reflect a rather short follow-up time of 12 months as both remission and transition rates are likely to increase over time [53].

The strengths of this study include a sizeable follow-up sample as well as clinical, functional, and cognitive measures assessed by trained raters as well as comprehensive MRS data. Limiting the study is, besides a—for UHR studies—short follow-up time, the lack of MRS data at 12-month follow-up. Likewise, is the known heterogeneity of outcome of UHR individuals [54] an inherent limitation to all UHR studies.

In conclusion, future studies should attempt to obtain longer follow-up times, as metabolic perturbances might be present in individuals that transition to a psychotic disorder after a longer period. This would better reflect long-term clinical outcome and uncover the possible underlying glutamatergic and GABAergic disturbances that might characterize UHR individuals who transition to a psychotic disorder. The considerable variation of findings across studies underlines the likely multifactorial influences on clinical outcome of UHR individuals and points toward the need for future studies combining different imaging modalities along with clinical, cognitive, and functional baseline measures to better predict long-term outcome. Future studies should also aim to include follow-up measures of glutamate and GABA levels to assess whether associations to cognitive levels are stable over time and whether metabolite levels are related to illness progression.

## Data Availability

The data that support the findings of this study are available from the corresponding author, CW, upon reasonable request.
